# Acute Hepatitis of Unknown Origin in Children: Analysis of 17 Cases Admitted to the Bambino Gesù Children’s Hospital in Rome

**DOI:** 10.3390/microorganisms12040826

**Published:** 2024-04-19

**Authors:** Velia Chiara Di Maio, Leonarda Gentile, Rossana Scutari, Luna Colagrossi, Luana Coltella, Stefania Ranno, Giulia Linardos, Daniela Liccardo, Maria Sole Basso, Andrea Pietrobattista, Simona Landi, Lorena Forqué, Marta Ciofi Degli Atti, Lara Ricotta, Andrea Onetti Muda, Giuseppe Maggiore, Massimiliano Raponi, Carlo Federico Perno, Cristina Russo

**Affiliations:** 1 Microbiology and Diagnostic Immunology Unit, Bambino Gesù Children’s Hospital, IRCCS, 00165 Rome, Italy; veliachiara.dimaio@opbg.net (V.C.D.M.);; 2 Multimodal Laboratory Research Unit, Bambino Gesù Children’s Hospital, IRCCS, 00165 Rome, Italy; 3 Hepatogastroenterology, Rehabilitative Nutrition, Digestive Endoscopy and Liver Transplant Unit, ERN RARE LIVER, Bambino Gesù Children’s Hospital, IRCCS, 00165 Rome, Italy; 4 Clinical Pathways and Epidemiology Unit, Bambino Gesù Children’s Hospital, IRCCS, 00165 Rome, Italy; 5 Medical Direction, Bambino Gesù Children’s Hospital, IRCCS, 00165 Rome, Italy; 6 Scientific Direction, Bambino Gesù Children’s Hospital, IRCCS, 00165 Rome, Italy

**Keywords:** acute hepatitis, adenovirus, pediatric

## Abstract

This study described 17 cases of children admitted to the Bambino Gesù Children’s Hospital with acute hepatitis of unknown origin between mid-April and November 2022. Following the World Health Organization’s working case definition of probable cases, 17 children, with a median age of 2.1 years (interquartile range: 1.0–7.1), presenting with acute hepatitis non-AE, with serum transaminase >500 IU/L, were included in the study. A pre-specified set of microbiological tests was performed on different biological specimens for all pediatric patients. All patients resulted negative for the common hepatotropic viruses. The most common pathogen detected in blood specimens was human-herpes-virus-7 (52.9%). Adenovirus was detected more frequently in stool specimens (62.5%) than in respiratory (20.0%) or blood samples (17.6%). Regarding Severe Acute Respiratory Syndrome Coronavirus 2 (SARS-CoV-2) infection, one child tested positive two days after admission, while antibodies against spike and nucleoprotein were present in 82.3% of patients. A co-pathogen detection was observed in 94.1% of children. Overall, 16 children recovered without clinical complications, while one patient required liver transplantation. In these cases of acute hepatitis of unknown origin, adenovirus was mainly detected in stool samples. A co-pathogen detection was also frequently observed, suggesting that the etiology of this acute hepatitis is most probably multifactorial.

## 1. Introduction

Since April 2022, an increase in severe acute hepatitis cases of unknown etiology among children with no underlying conditions has been reported in various countries [1]. Initially, cases were predominantly identified in the UK, but other countries began reporting similar events soon after. In Italy, a survey study was conducted by the liver group of the Italian Society of Pediatric Gastroenterology, Hepatology and Nutrition (SIGENP) between March and May 2022, reporting 15 cases with a severe acute hepatitis without any putative cause [2]. The World Health Organization reported a total of 1010 probable cases from 35 different countries worldwide between April and July 2022 [3]. The majority were children aged from 1 month to 16 years old. Most of the pediatric cases were hospitalized and showed typical manifestations of acute hepatitis including vomiting, diarrhea, jaundice, and a high level of liver enzymes. Investigation on common viruses that cause acute viral hepatitis (A, B, C, D, and E) resulted negative. Progression to acute liver failure was also reported and 46 children required liver transplant and 22 died [3]. To date, the etiology of these acute hepatitis cases in children remains unknown. However, working hypotheses involving a link to viral infection have been proposed. In particular, adenovirus infection due to abnormal susceptibility; host response; or abnormal susceptibility resulting from previous infection, co-infection with severe acute respiratory syndrome coronavirus 2 (SARS-CoV-2), or other pathogens have been reported [1]. Other possible viral causes involve a novel variant of adenovirus or SARS-CoV-2, a novel pathogen or a post-infectious SARS-CoV-2 syndrome. Furthermore, recent data showed that adeno-associated virus 2 (AAV2) was frequently detected using metagenomic analysis in samples from children with acute hepatitis of unknown origin [4,5,6]. Starting with this evidence, a new working hypothesis, which involves a combination of AAV2 infection in the presence of a helper virus (probably adenovirus F41 or human herpes virus 6 [HHV-6]), has been proposed [7]. In addition, a non-infective etiology due to drug, toxin, or environmental exposure has been suggested [1]. This study describes 17 cases of children admitted to the Bambino Gesù Children’s Hospital Healthcare and Research Institute in Rome with acute hepatitis of unknown origin between mid-April and November 2022. All pediatric patients were followed up with a predefined methodological approach including the same microbiological tests on multiple biological specimens.

## 2. Materials and Methods

### 2.1. Patients

According to the WHO working case definition [3], 17 children who were admitted to Bambino Gesù Children’s Hospital between mid-April 2022 and November 2022 presenting with acute hepatitis non-AE (hepAE) with serum transaminase > 500 IU/L (alanine aminotransferase [ALT] and/or aspartate aminotransferase [AST]), who were 16 years old or younger, were considered as probable cases. Demographic and clinical data were collected anonymously for all patients.

### 2.2. Laboratory Testing

A pre-specified set of microbiological tests was planned and performed for all children on multiple biological specimens (blood, stool, and respiratory samples) (Table 1). All laboratory investigations were performed following the recommendations from the European Center for Disease Prevention and Control (ECDC) for diagnostic testing in the suspected cases of acute non-hep AE [8].

#### 2.2.1. Nucleic Acid Amplification Tests (NAATs) and Antigen Test

Nucleic acids (DNA/RNA) were extracted from various specimen types (whole blood, plasma samples, pre-treated stool samples, nasopharyngeal swab, or nasopharyngeal aspirate) using two different automatic platforms—QIAsymphony (Qiagen, Hilden, Germany) and/or Seegene STARlet—following the manufacturer’s instructions. For DNA/RNA extracted from whole blood or plasma using QIAsymphony (Qiagen), a real-time polymerase chain reaction (RT-PCR) was performed using a number of commercial kits to detect adenovirus (Adenovirus R-gene kit, BioMérieux, Marcy-l’Étoile, France) including all known human adenovirus serotypes), cytomegalovirus (CMV; Artus CMV TM PCR kit, Qiagen), enteroviruses (Enterovirus R-gene kit, BioMérieux), Epstein–Barr virus (EBV; Artus EBV TM PCR kit, Qiagen), hepatitis A virus (HAV; Quanty HAV kit, Clonit, Milan, Italy), hepatitis B virus (HBV; Artus HBV QS-RGQ kit, Qiagen), hepatitis C virus (HCV; Xpert HCV Viral Load, Cepheid, Sunnyvale, CA, USA), hepatitis D virus (HDV; Quanty HDV kit, Clonit), hepatitis E virus (HEV; HEV RT-PCR kit, Altona Diagnostics, Hamburg, Germany), herpes simplex virus 1 and 2 (HSV-1/2; HSV-1/2 R-gene kit, BioMérieux), human herpes virus 6 and 7 (HHV-6 R-gene kit, BioMérieux; HHV-7 Elite MGB kit, ELITech Group, Turin, Italy), and parvovirus B19 (PB19; Artus PB19 TM PC kit, Qiagen). All the PCR reactions included internal controls. Standards, as well as external positive and negative controls were run in parallel. A calibration curve for quantitation was created using the standards that were supplied. For each protocol, the limit of detection and the limit of quantification were as reported by the manufacturer. Quantitative results were expressed as viral copies/mL. Nucleic acids extracted from stool samples using the Seegene STARlet platform were amplified using the Allplex Gastrointestinal Panel Assays (Seegene, Seoul, Republic of Korea), including the most common viruses and bacteria causing gastrointestinal tract infections (see Table 1). Moreover, for stool samples, RT-PCR was also performed to detect enteroviruses, human parechovirus (using Enterovirus R-gene kit and Parechovirus R-gene kit, Biomérieux), and adenovirus (using Adenovirus R-gene kit, Biomérieux). Nucleic acids extracted from nasopharyngeal swabs or nasopharyngeal aspirates using the Seegene STARlet platform were amplified using the Allplex Respiratory Panel Assays (Seegene, Seoul, Republic of Korea), including several viruses involved in respiratory tract infections (see Table 1). In the interpretation of the results of the Allplex Gastrointestinal and Respiratory Panel assays, the sample was considered positive for the presence of the pathogen if the Cycle threshold (Ct) value was ≤42 cycles. A negative specimen was defined by the absence of amplification for any pathogen, with the presence of amplification of the internal control. 

The presence of SARS-CoV-2 was assessed using antigenic or molecular tests. SARS-CoV-2 antigen testing was performed using the electrochemiluminescence immunoassay (ECLIA) Roche Elecsys SARS-CoV-2 Antigen on Roche Cobas e411 (Roche Diagnostics GmbH, Mannheim, Germany). According to the manufacturer’s instructions, a result of cut off index (COI) ≥ 1.0 was interpreted as reactive for the SARS-CoV-2 antigen.

The molecular test was assessed using The Cepheid Xpert Xpress CoV-2 plus performed on Cepheid’s GeneXpert^®^DX system (Cepheid, Sunnyvale, CA, USA).

#### 2.2.2. Serologic Tests

Serologic testing was performed on serum samples for the detection of immunoglobulin G (IgG) and/or immunoglobulin M (IgM) of CMV, HDV, HEV, human immunodeficiency virus (HIV), HSV-1/2, PB19, and varicella, through Chemiluminescence Immunoassay (CLIA) using LIAISON^®^ XL system (DiaSorin, Saluggia, Italy), following the manufacturer’s instructions. Specific antibodies (IgG and IgM) against EBV viral capsid antigen (VCA), IgG antibodies to Epstein–Barr Nuclear Antigen-1 (EBNA-1), serological markers associated with HBV (hepatitis B surface antigen [HBsAg], antibody to HBsAg [anti-HBs], hepatitis B core antigen [HBcAg], antibody to HBcAg [anti-HBc], hepatitis B e antigen [HBeAg], antibody to HBeAg [anti-HBe]), and antibodies (IgG and IgM) against hepatitis A and C viruses were detected using the Chemiluminescent Microparticle Immunoassay (CMIA) with the Alinity system (Abbott Laboratories, Abbott Park, IL, USA). The presence of anti-HHV-6 and anti-adenovirus IgG and IgM was evaluated through commercial ELISA kits (Euroimmun, Lubeka, Germany). Detection of SARS-CoV-2 anti-spike (S) and anti-nucleoprotein (N) was performed with electrochemiluminescence immunoassay (ECLIA; Roche, Elecsys SARS-CoV-2 anti-N total and Roche, Elecsys, Anti-SARS-CoV-2 S total) using Cobas e411 (Roche Diagnostics GmbH, Mannheim, Germany). All the tests were performed following the manufacturer’s recommendations and the results were interpreted using the manufacturer’s cut-off values.

### 2.3. Statistical Analysis

Descriptive statistics are expressed as median values and interquartile range (IQR) for continuous data and number (percentage) for categorical data. Statistical comparisons were performed using Fisher’s exact test. A value of *p* less than 0.05 was considered statistically significant.

## 3. Results

### 3.1. Demographics and Clinical Characteristics

Between mid-April 2022 and November 2022, 17 children with acute hepatitis of unknown origin were admitted to the Bambino Gesù Children’s Hospital Healthcare and Research Institute in Rome. The median age of children was 2.1 years (interquartile range [IQR]: 1.0–7.1) and 10 (58.8%) of them were male (Table 2). All children were born in Italy, 11 [64.7%] came from Central Italy, 5 [29.4%] from the South, and 1 [5.9%] from the North. All were immunocompetent and no significant comorbidities were reported for any of them. At hospital admission, all the pediatric patients showed high levels of liver enzymes ALT 801 U/L (IQR: 616–1163); AST 489 U/L (IQR: 315–1011); and lactate dehydrogenase [LDH] 518 U/L (IQR: 417–665, see Table 2). Initial reported symptoms included fever (N = 9, 52.9%), followed by diarrhea (N = 5, 29.4%), vomiting (N = 6, 35.3%), jaundice (N = 4, 23.5%), and respiratory symptoms (N = 4, 23.5%). The median time from symptom onset to hospital admission was 12.0 days (IQR 3.0–10.0). The median time of hospitalization was 9.0 days (IQR: 5.8–16.8). Of these patients, 16 (94.1%) recovered without clinical complications, while 1 patient underwent a liver transplant (Table 2).

**Figure 1:**
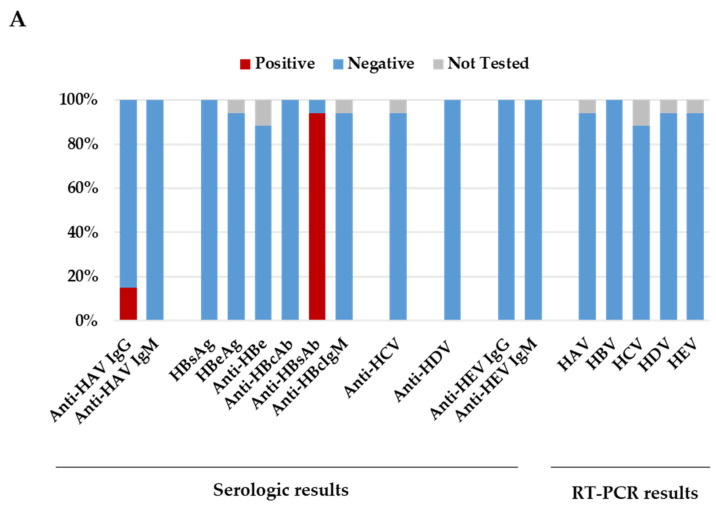

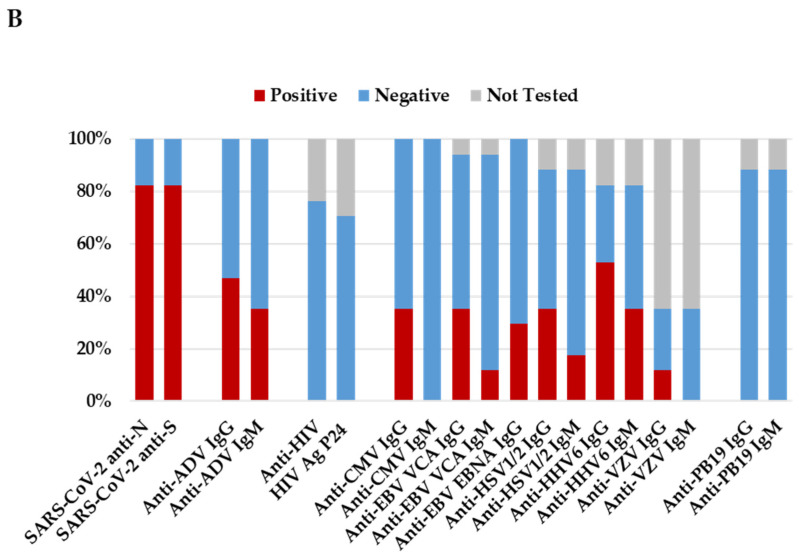
Results related to laboratory investigations performed following the recommendations from the European Center for Disease Prevention and Control (ECDC). The graph in (**A**) shows serologic and RT-PCR results related to the investigation on common viruses that cause acute viral hepatitis (hepatitis viruses A, B, C, D, and E). The graph in (**B**) shows results related to serology for all the other viruses investigated.

None of the pediatric patients died. All 17 patients were tested for the common viruses that cause acute viral hepatitis (A, B, C, D, and E) and resulted negative (Figure 1A). 

### 3.2. Laboratory Investigation Results

A whole set of additional molecular and serologic tests was also performed for all patients in different specimen types (whole blood, serum, stool, nasopharyngeal aspirate, or nasopharyngeal swab). All patients were tested for all the pathogens included in the laboratory investigations, considering the sample’s availability.

**Figure 2:**
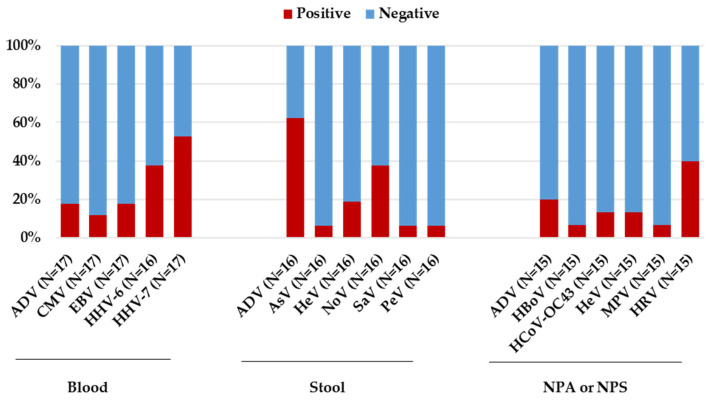
Results related to laboratory investigations performed in different specimen typed (blood, stool, nasopharyngeal aspirate, or nasopharyngeal swab) using real-time PCR test for the 17 patients with acute hepatitis of unknown origin. The prevalence of positive and negative samples was calculated on the total of samples tested for each pathogen.

Overall, using PCR testing, HHV-7 and HHV-6 were the most frequently detected viruses in blood, with 9/17 (52.9%) and 6/16 (37.5%) children testing positive, respectively (Table 3, Figure 2).

The viral load range of HHV-7 was below the limit of quantification to 27,300 copies/mL, while for HHV-6, the viral load range was below the limit of quantification to 3837 copies/mL. Other herpetic viruses, EBV and CMV, were detected in 3/17 (17.6%) and 2/17 (11.8%) of the children, respectively (Table 3). For all these viruses, IgM was detected only in a few patients (anti-EBV VCA IgM, 2/16 [12.5%]; anti-HHV-6 IgM, 5/14 [35.7%]; Figure 1B). In the stool specimens, adenovirus was the most frequently detected virus with 10/16 (62.5%) children testing positive, followed by norovirus and enterovirus detected in 6/16 (37.5%) and 3/16 (18.7%) children, respectively (Table 3). In respiratory samples, rhinovirus was the most frequently detected pathogen in 6/15 (40.0%) children, followed by adenovirus detected in 3/15 (20.0%) children. In particular, adenovirus detection was more common in stool specimens (10/16, 62.5%) than in respiratory samples (3/15, 20.0%) or blood specimens (3/17, 17.6%, Table 3). Considering positive samples, the median human adenovirus viral load on PCR was 4822 copies/mL (range—below the limit of quantification to >100,000,000 copies/mL) in stool specimens, while was 5310 copies/mL (range—3330–7399 copies/mL) in blood specimens. Furthermore, of the 10 adenovirus positive stool samples detected using quantitative RT-PCR (which targets all known human adenovirus serotypes), three (30.0%) tested positive with the gastrointestinal multiplex PCR panel, which specifically detects adenovirus serotypes F40-41. Concerning the serologic results, adenovirus IgG antibodies were detected in 8/17 (47.1%) patients, while IgM antibodies were detected in 6/17 (35.3%) patients (Figure 1B). Two patients, with adenovirus-positive RT-PCR (one in the blood and the other in the stool sample) were both also positive for IgG and IgM. Regarding SARS-CoV-2 infection, an antigen or PCR test was performed in all children at hospital admission with negative results. However, one child tested positive two days after admission. To investigate a possible prior SARS-CoV-2 exposure, serum samples were analyzed for antibodies against SARS-CoV-2 spike and nucleoprotein. Interestingly, 14/17 (82.3%) children tested positive for both SARS-CoV-2 anti-S and anti-N (Figure 1B). Notably, 9 out of 12 (75.0%) children with adenovirus infection tested positive for anti-S and anti-N SARS-CoV-2 antibodies. By performing a case-by-case analysis for co-pathogen detection, 16/17 (94.1%) children had a coinfection with at least one other pathogen (Table S1). Of the five children who tested negative for adenovirus, four showed a coinfection with at least two pathogens. In particular, one tested positive for EBV, HHV-6, HHV-7 in the whole blood sample, parechovirus and norovirus in the stool sample, and enterovirus/rhinovirus and metapneumovirus in the nasopharyngeal aspirate. Another patient showed EBV and HHV-6 in the whole blood sample. The last two patients showed, respectively, HHV-7 in the whole blood sample and *Clostridium difficile* in the stool sample, while the other patient showed HHV-6 and HHV-7 in the whole blood sample; parechovirus, norovirus, and enteroaggregative *Escherichia coli* in the stool sample; and rhinovirus in the respiratory sample. Co-infecting pathogens in the 12 adenovirus positive patients included herpes viruses, detected in 9/12 (75.0%, HHV-7 N = 6; HHV-6 N = 3; CMV N = 2 and EBV N = 1); enteric pathogens, detected in 8/12 (66.7%, enterovirus N = 3; norovirus N = 4; astrovirus N = 1, Aereomonas N = 2, *Clostridium difficile* N = 2, sapovirus N = 1); and respiratory pathogens, detected in 7/11 pediatric patients (63.6%, human Coronavirus OC43 N = 2, human rhinovirus N = 5, bocavirus N = 1, and enterovirus N = 1). By analyzing the frequencies of these coinfections, no evidence of a significant correlation of liver pathology with specific virus–virus coinfection was found, independent of the biological specimen (blood, respiratory samples, and stool) under study.

### 3.3. Analysis of HHV-6 and HHV-7 Prevalence 

Since a high frequency of HHV-6 and HHV-7 was observed in blood samples of the pediatric patients with unknown hepatitis (Table 3 and Figure 2), we also analyzed the prevalence of these two viruses in blood samples taken from the pediatric population aged 0 to 16 years admitted to our hospital in the same study period. Overall, no significant differences in prevalence were observed for HHV-6 and HHV-7 between the general pediatric population and the children with hepatitis of unknown origin (for HHV-6: 47.9% [465/970] vs. 37.5% [6/16], *p*-value = 0.46; for HHV-7: 46.7% [106/227] vs. 52.9% [9/17], *p*-value = 0.80).

## 4. Discussion

Recently, several clusters of acute hepatitis of unknown origin have been reported in children worldwide. Between mid-April 2022 and November 2022, 17 children with severe hepatitis of unknown origin were admitted to the Bambino Gesù Children’s Hospital Healthcare and Research Institute in Rome. All showed clinical and epidemiological characteristics linked to the definition proposed by the WHO of a probable case of acute hepatitis of unknown origin [3]. So far, the etiology of these reported cases worldwide remains unknown. Currently, the main working hypothesis involves a direct role of one or more pathogens. Therefore, it is important to perform a methodologically correct and complete panel of screening tests for all probable hepatitis cases, in order to evaluate the possible role of an etiological agent. One strength of this study is that a pre-established diagnostic protocol, including a wide range of molecular and serologic tests performed in different specimen types (blood, stool, nasopharyngeal aspirate, or nasopharyngeal swab), was followed for all patients in parallel. Among all the working hypotheses that have been investigated, one involves the direct role of adenovirus infection [1,3], since this is the most frequent pathogen detected in various samples [9,10,11,12,13]. Indeed, as reported in the latest available updates, around 52% of the cases reported by the ECDC, 66% reported from the UK cohort and 45% by the Center for Disease Control (CDC), tested positive for adenovirus in one or more biological specimen [12,13,14]. In most cases, adenovirus was detected more frequently in blood or serum samples than in stool or respiratory samples [11,12]. Among the cases considered in this study, adenovirus was detected in 70.6% of them, in at least one specimen type. Different from other published data, we observed a high rate of adenovirus in stool samples (62.5%) compared to blood samples (20.0%). Interestingly, this rate of adenovirus-positive stool samples was higher when compared to that detected in the stool samples of children aged 0 to 16 years admitted to our hospital in the same period (62.5%, 10/16 vs. 33.0%, 216/655 adenovirus-positive stool samples; *p*-value = 0.03). A recent study reported by the United Kingdom showed an increase in the prevalence of adenovirus-positive stool samples in children aged 1–4 years compared to pre-pandemic levels [1]. The observed increase in adenovirus circulation could also explain the high rate of adenovirus positive samples detected among children with unknown hepatitis. Case control studies are currently lacking to clarify this observation [7]. Concerning the serologic results, 6 out of 12 children with a positive adenovirus molecular test (in at least one specimen type) showed anti-adenovirus IgM (two of them also had an IgG-positive result). Of the other patients, five had IgG-positive and IgM-negative results, while only one pediatric patient had both IgG- and IgM-negative results (probably indicating a very recent infection). Determination of specific antibodies using anti-adenovirus serologic tests can corroborate the likelihood of acute infection, especially if IgM class antibodies are present, but also in the presence of IgG. A negative serologic test cannot rule out the presence of an infection, especially in the early stages when IgM antibodies may not yet have been produced or are present at very low levels that they cannot be detected using serologic assays. For this reason, the serologic results (particularly those related to IgM) are not sufficient to arrive at a diagnosis of acute adenovirus infection, but should always be evaluated in the context of the case history and also evaluated through other laboratory tests (e.g., molecular tests). Human adenoviruses are divided into seven species (A–G) and, to date, 111 genotypes have been identified and characterized using whole genome analysis (http://hadvwg.gmu.edu/, accessed on 27 February 2024). Different adenovirus serotypes exhibit different tissue tropisms and clinical manifestations of infection [15]. Adenovirus infections are frequent during childhood. Commonly, adenoviruses are responsible for diseases in the respiratory, ocular, gastrointestinal, and renal tracts. Instead, acute hepatitis is an uncommon manifestation of adenovirus infection and is mainly observed in immunocompromised children [16,17]. The role of this virus in the development of acute hepatitis in immunocompetent children has rarely been reported in the past [18,19,20]. To characterize the subtypes detected in the adenovirus-positive samples of children with unknown hepatitis, partial sequencing of the hexon gene was performed in some samples of the cases reported from the UK and US. In particular, it was found that adenovirus serotype F41 was the most frequently detected (77% of cases with available data from the UK, 55% of the initial cluster reported in Alabama; 59% of cases with available data from the US; 45% reported by the ECDC) [9,12,14]. This serotype is a common cause of gastroenteritis in young children across the world, but no previous evidence of the association with liver inflammation has been reported. No molecular characterization of positive adenovirus samples was carried out in this study. However, by using the multiplex PCR gastrointestinal system (that specifically detects adenovirus serotypes F40-41), we were able to determine serotype in the adenovirus-positive stool samples (N = 10). Three out of ten patients tested positive for adenovirus F40-41 (30.0%). This prevalence is significantly lower compared to that observed in cases reported from other countries. Even though laboratory and epidemiological data may suggest a possible role of adenovirus in the development of these cases of acute hepatitis, the available liver histologic data showed no evidence of viral inclusions or particles [9]. Furthermore, a survey reported by the CDC showed no increase in pediatric hepatitis or adenovirus serotype F40-41 above baseline levels observed before the COVID-19 pandemic [21]. Overall, these data suggest that the detection of adenovirus from stool or blood is not sufficient to link this agent, alone, to the development of unexplained forms of acute severe hepatitis in children. Rather, the additional possible pathogenic mechanisms proposed suggest the role of co-infection with other pathogens or infection with SARS-CoV-2 (prior or current) as a trigger in the development of acute hepatitis. Regarding the hypothesis involving SARS-CoV-2 as a co-factor, a recent published correspondence showed the possible role of SARS-CoV-2 infection, since the virus could persist within the gastrointestinal tract [22]. It has been hypothesized that these pediatric cases with severe acute hepatitis could be a consequence of adenovirus infection with intestinal tropism in children previously infected by SARS-CoV-2 and carrying viral reservoirs [23,24]. Available data from EA/EUU has shown a low prevalence on active or recent SARS-CoV-2 infection in children affected by acute hepatitis (40/392, 10.2% tested positive for SARS-CoV-2). In addition, in this population, none of the patients showed an active SARS-CoV-2 infection at hospital admission. However, 75.0% of patients with positive adenovirus samples showed anti-N and anti-S antibodies, thus indicating that the majority had been previously exposed to SARS-CoV-2. We also performed a retrospective evaluation of the overall prevalence of anti-N and anti-S in the pediatric population aged from 0 to 16 years old admitted to the Bambino Gesù Children’s Hospital in Rome starting from April 2022. Notably, 961 out of 1287 (74.7%) patients, with an available serologic test, resulted positive for both SARS-CoV-2 anti-N and anti-S. Thus, there was no significant difference in anti-N and anti-S seroprevalence between our positive adenovirus cases with a previous SARS-CoV-2 exposure and the general community setting of the same age admitted to our hospital. Currently, few data are available from other countries regarding SARS-CoV-2 seroprevalence. In the latest update of the ECDC, serologic results were only available for 115 cases, of which 73 (63.5%) resulted positive [12]. However, whether a past or current SARS-CoV-2 infection is crucial in the progression of hepatitis in the contest of adenovirus coinfection remains controversial. In addition to adenovirus, in these cases, other pathogens were also detected, most commonly HHV-6 and HHV-7 in blood, and rhinovirus in respiratory samples. Other studies have also reported the detection of these viruses [2,4,8]. Concerning HHV-6 and 7, these viruses typically cause primary infections in children and adolescents usually with mild symptoms, while acute liver failure is rarely documented [25,26]. We also analyzed the prevalence of HHV-6 and HHV-7 in blood samples taken from the pediatric population aged 0 to 16 years admitted to our hospital in the same study period. No significant differences in prevalence were observed for HHV-6 and HHV-7 between the general pediatric population and the children with hepatitis of unknown origin. Regarding other herpetic viruses, by evaluating serologic results, a low prevalence of IgM-positive samples was observed for HSV-1/2 and EBV, although with a negative PCR test. It should be pointed out that the specificity of this class of antibodies is low, particularly for the herpetic viruses. In fact, the presence of IgM antibodies may represent either recent infection or viral reactivation, or even a latent or persistent subclinical infection. For this reason, serologic results alone are not sufficient to define herpetic infection. Additionally, the detection of Rhinovirus in respirator samples is in line with the frequency found in the general child population admitted to our hospital. As reported by other studies, these results are difficult to interpret and the role of these viruses as possible etiological agents of acute hepatitis and liver failure remains unclear [7]. Interestingly, in this study we observed that 94.1% of probable cases showed a co-infection with at least one other pathogen. Co-infections mainly involved respiratory viruses and enteric pathogens. Common findings regarding co-pathogen detection were observed in other European and American cases of acute hepatitis of unknown origin recently reported [9,24]. One possible explanation has been attributed to an increased susceptibility of immune response in immune-naïve young children, due to limited exposure to respiratory and enteric viruses that circulated at lower frequencies during the last two years of the COVID-19 pandemic, as a result of lockdown measures [27]. Therefore, a reasonable hypothesis is that simultaneous or consecutive viral infections could trigger severe hyperinflammatory responses in the liver, most probably in previously immunologically naïve children, who have a particular genetic background [7]. Moreover, three recently published studies (two from the UK and the other from the USA), using a metagenomic approach, provided evidence that infection by AAV2 is linked to cases of acute hepatitis of unknown origin [4,5,6]. Findings reported in these papers hypothesize that cases of acute hepatitis of unknown origin could be a result of various factors including AAV2, a helper virus (such as adenovirus or HHV-6), and genetic predisposition [4,5,6]. However, this hypothesis is still under investigation. These results highlight the potential and the utility of a metagenomic approach to investigate diseases with unknown etiology, such as these cases of acute hepatitis of unknown origin in children. This technique is based on the sequencing of all DNA and/or RNA found in a clinical sample, thus opening up the opportunity to investigate all possible microbiological agents present. As metagenomic analysis requires specific expertise, it is not yet used for routinely clinical diagnostics and is only carried out by a small number of laboratories. 

## 5. Conclusions

Taken together, the observations reported in this study do not support the hypothesis of a unique agent as a potential etiological factor for this hepatitis. They, rather, suggest that the etiology of this acute hepatitis could potentially be related to the co-presence of multiple viral infections able to activate hyperinflammatory phenomena that, in turn, cause liver damage. Further studies are warranted to expand the knowledge in this setting.

## Figures and Tables

**Table 1:** Specimen type for testing and laboratory investigations performed.

	Pathogens
Blood (whole or serum)	
Serology	ADV, CMV, EBV, HAV, HBV, HCV, HDV, HEV, HHV-6, HIV, HSV-1/2, PB19, SARS-CoV-2 anti-S and anti-N, VZV
NAAT	ADV, CMV, EBV, HAV, HBV, HCV, HDV, HEV, HeV, HHV-6 and 7, HSV-1/2, PB19
Nasopharyngeal swab or nasopharyngeal aspirate	
NAAT	Respiratory viruses screening by multiplex panel assay (including ADV, Flu A, Flu B, HBoV, HCoV-229E, HCoV-NL63, HCoV-OC43, HeV, HRV, PIV 1,2,3 and 4, RSV A and B) SARS-CoV-2
Antigen	SARS-CoV-2
Stool	
NAAT	Enteric viruses and bacteria screening by multiplex panel assay (including ADV, Aer, AstV, Cam, CD hyper, CdB, *E. coli* O157, EAEC, EHEC, EPEC, ETEC, NoV-GI and GII, RotV, Sal, EIEC/Sh, SaV, Yer) HPeV, HeV

**Table 2:** Demographics and clinical characteristics of the 17 pediatric patients with acute hepatitis of unknown origin.

Pediatric patients, N	17
Gender, N (%)	
Male	10 (58.8)
Female	7 (41.2)
Age (years), Median (IQR)	2.1 (1.0–7.1)
Liver function index U/L, Median (IQR)	
ALT	801 (616–1163)
AST	489 (315–1011)
LDH	518 (417–665)
Clinical sign/symptoms, N (%)	
Fever	9 (52.9)
Diarrhea	5 (29.4)
Vomiting	6 (35.3)
Jaundice	4 (23.5)
Upper respiratory symptoms ^†^	4 (23.5)
Length of hospitalization (days), Median (IQR)	9.0 (5.8–16.8)
Outcome, N (%)	
Hospital discharge without clinical complications	16 (94.1)
Liver Transplantation	1 (5.9)
Death	0 (0.0)

^†^ Upper respiratory symptoms included dry cough and bronchitis.

**Table 3:** Pathogens detected in different sample types using real-time PCR tests for the 17 patients with acute hepatitis of unknown origin.

Type of Sample	Pathogen	No. Positive Tests/ No. of Total Specimens Tested (%)
Blood		
	Adenovirus group ^†^	3/17 (17.6)
	Cytomegalovirus	2/17 (11.8)
	Epstein–Barr Virus	3/17 (17.6)
	Human herpesvirus 6	6/16 (37.5)
	Human herpesvirus 7	9/17 (52.9)
Stool		
	Adenovirus	10/16 (62.5)
	Astrovirus	1/16 (6.2)
	Enterovirus	3/16 (18.7)
	Norovirus	6/16 (37.5)
	Sapovirus	1/16 (6.2)
	Parecovirus	1/16 (6.2)
Nasopharyngeal aspirate or nasopharyngeal swab		
	Adenovirus	3/15 (20.0)
	Bocavirus	1/15 (6.7)
	Coronavirus OC43	2/15 (13.3)
	Enterovirus	2/15 (13.3)
	Metapneumovirus	1/15 (6.7)
	Rhinovirus	6/15 (40.0)

^†^ Including all known human adenovirus serotypes.

## Data Availability

Data are contained within the article (and Supplementary Materials).

## Supplementary Materials

The following supporting information can be downloaded at: https://www.mdpi.com/article/10.3390/microorganisms12040826/s1, Table S1: Detailed results of co-pathogen detection reported for each patient in different specimen types.

## Abbreviations

AdV: adenovirus; ALT: alanine aminotransferase; AST: aspartate aminotransferase; AstV: astrovirus; Cam: *Campylobacter* spp.; CD hyper: hypervirule *Clostridium difficile*; CdB: *Clostridium difficile* toxin B; CMV: cytomegalovirus; *E. coli* O157: *Escherichia coli* O157; EAEC: enteroaggregative *Escherichia coli*; EBV: Epstein–Barr virus; EHEC: Enterohemorrhagic *Escherichia coli*; EIEC/Sh: Enteroinvasive *Escherichia coli*/Shighella; EPEC: enteropathogenic *Escherichia coli*; ETEC: enterotoxigenic *Escherichia coli*; Flu A: influenza A; Flu B: influenza B; HAV: hepatitis A virus; HBoV: human Bocavirus 1/2/3/4; HBV: hepatitis B virus; HCoV-229E: human coronavirus 229E; HCoV-NL63: human coronavirus NL63; HCoV-OC43: human coronavirus OC43; HCV: hepatitis C virus; HDV: hepatitis D virus; HeV: enterovirus; HEV: hepatitis E virus; HHV-6: human herpesvirus 6; HHV-7: human herpesvirus 7; HIV: human immunodeficiency virus; HRV: human rhinovirus; HSV-1/2: herpes simplex virus type 1 and 2; IQR: interquartile range; LDH: lactate dehydrogenase; NAAT: nucleic acid amplification test; NoV-GI and GII: norovirus GI and GII; PB19: parvovirus B19; PeV: parechovirus; PIV 1,2,3,4: parainfluenza viruses 1, 2, 3, and 4; RotV: rotavirus; RSV A and B: respiratory syncytial virus A and B; Sal: *Salmonella* spp.; SARS-CoV-2 anti-S and anti-N: SARS-CoV-2 (severe acute respiratory syndrome coronavirus-2) anti-spike (S) and anti-nucleoprotein; (N); SARS-CoV-2: severe acute respiratory syndrome coronavirus 2; SaV: sapovirus; Vib: *Vibrio* spp.; VZV: varicella-zoster virus; Yer: yersinia enterocolitica.
