# Supplementary material for: Acute Hepatitis of Unknown Origin in Children: Analysis of 17 Cases Admitted to the Bambino Gesù Children’s Hospital in Rome
Source: Microorganisms. 2024 Apr 19;12(4):826. doi: 10.3390/microorganisms12040826 (PMC11051986; doi:10.3390/microorganisms12040826)
Supplement: Supplementary file 1 [file microorganisms-12-00826-s001.zip › microorganisms-2924733-supplementary.docx]

| **Type**  **of**  **sample** | **Pathogen** | **PT1** | **PT2** | **PT3** | **PT4** | **PT5** | **PT6** | **PT7** | **PT8** | **PT9** | **PT10** | **PT11** | **PT12** | **PT13** | **PT14** | **PT15** | **PT16** | **PT17** |
| --- | --- | --- | --- | --- | --- | --- | --- | --- | --- | --- | --- | --- | --- | --- | --- | --- | --- | --- |
| **Blood** | **AdV** |  |  |  |  |  |  |  |  |  |  |  |  |  |  |  |  |  |
|  | **CMV** |  |  |  |  |  |  |  |  |  |  |  |  |  |  |  |  |  |
|  | **EBV** |  |  |  |  |  |  |  |  |  |  |  |  |  |  |  |  |  |
|  | **HHV-6** |  |  |  |  |  |  |  |  |  |  |  |  |  |  |  |  |  |
|  | **HHV-7** |  |  |  |  |  |  |  |  |  |  |  |  |  |  |  |  |  |
| **Stool** | **AdV** |  |  |  |  |  |  |  |  |  |  |  |  |  |  |  |  |  |
|  | **Aer** |  |  |  |  |  |  |  |  |  |  |  |  |  |  |  |  |  |
|  | **AstV** |  |  |  |  |  |  |  |  |  |  |  |  |  |  |  |  |  |
|  | **CdB** |  |  |  |  |  |  |  |  |  |  |  |  |  |  |  |  |  |
|  | **HEV** |  |  |  |  |  |  |  |  |  |  |  |  |  |  |  |  |  |
|  | **HPeV** |  |  |  |  |  |  |  |  |  |  |  |  |  |  |  |  |  |
|  | **SV** |  |  |  |  |  |  |  |  |  |  |  |  |  |  |  |  |  |
|  | **NoV** |  |  |  |  |  |  |  |  |  |  |  |  |  |  |  |  |  |
|  | **EAEC** |  |  |  |  |  |  |  |  |  |  |  |  |  |  |  |  |  |
|  | **EPEC** |  |  |  |  |  |  |  |  |  |  |  |  |  |  |  |  |  |
| **NPA**  **or**  **NPS** | **AdV** |  |  |  |  |  |  |  |  |  |  |  |  |  |  |  |  |  |
|  | **HCoV-OC43** |  |  |  |  |  |  |  |  |  |  |  |  |  |  |  |  |  |
|  | **HBoV** |  |  |  |  |  |  |  |  |  |  |  |  |  |  |  |  |  |
|  | **HEV** |  |  |  |  |  |  |  |  |  |  |  |  |  |  |  |  |  |
|  | **HRV** |  |  |  |  |  |  |  |  |  |  |  |  |  |  |  |  |  |
|  | **HEV/HRV** |  |  |  |  |  |  |  |  |  |  |  |  |  |  |  |  |  |
|  | **MPV** |  |  |  |  |  |  |  |  |  |  |  |  |  |  |  |  |  |
| Number of pathogens detected | | **7** | **5** | **4** | **3** | **5** | **2** | 7 | **4** | **2** | **4** | **2** | **3** | **2** | **6** | **0** | 5 | **4** |

Supplementary Table S1. Detailed results of co-pathogen detection reported for each patient in different specimen type.

**Adenovirus Positive samples**

**Test Not performed**

**Pathogen Not detected**

**Pathogen Detected**
